# The impact of acute weight loss following bariatric surgery on Eustachian tube function

**DOI:** 10.25122/jml-2023-0254

**Published:** 2023-09

**Authors:** Khalid Alyahya, Abdullah Alarfaj, Abdulelah AlBahr, Sarah AlBahar, Majd Alsaleh, Fahimah Almuhaytib, Abdulwahab Alyahya

**Affiliations:** 1 Otolaryngology, Head and Neck Surgery Department, King Faisal University, Hofuf, Saudi Arabia; 2 Surgery Department, King Fahad Hospital, Hofuf, Saudi Arabia; 3 Otolaryngology, Head and Neck Surgery Department, King Fahad Hospital of University, Al-Khobar, Saudi Arabia

**Keywords:** bariatric surgery, Eustachian Tube Dysfunction, obesity, ASMBS/IFSO: American Society of Metabolic and Bariatric Surgery/International Federation for the Surgery of Obesity and Metabolic Disorders, BS: Bariatric Surgery, BMI: Body Mass Index, DSVE: Dynamic Slow-Motion Video Endoscopy, ET: Eustachian Tube, ETDQ-7: Eustachian Tube Dysfunction Questionnaire-7, LVPM: Levator Veli Palatine Muscle, PET: Patulous Eustachian Tube, RYGB: Roux-en-Y Gastric Bypass, SG: Sleeve Gastrectomy, TVPM: Tensor Veli Palatini Muscle

## Abstract

Obesity has emerged as a pressing concern in contemporary society, prompting an increase in bariatric surgery (BS) procedures for severe obesity management. Post-bariatric weight loss might cause complications, such as a reduction in the soft tissue surrounding the Eustachian tube, potentially affecting its function. This cohort prospective study, conducted between May and December 2022, aimed to assess the impact of post-bariatric acute weight loss on Eustachian tube function. A total of 54 cases of bariatric surgery and 157 control subjects were included in the study. Data on socio-demographics, weight, and the type of bariatric surgery were collected for the study group. ET function was assessed using the Eustachian Tube Dysfunction Questionnaire (ETDQ-7). Approximately 55% of the participants fell within the age range of 18-25 years, with the majority (91.4%) having not undergone bariatric surgery. Conversely, participants who underwent BS were significantly more prevalent in the older age groups, specifically those over 50, between 40-50, and 31-40 years (p<0.001). Our sample consisted of 82.5% females and 17.5% males, with BS being significantly more common among male subjects (45.9%) compared to females (21.3%) (p=0.002). The mean total ETDQ-7 in control subjects was significantly higher (11.29±5.49) compared to those who had BS (9.11±4.09). Moreover, when comparing the ETDQ-7 between subjects who had BS and the control group, no statistically significant differences were observed in the total ETDQ-7 score and across all seven items within the ETDQ-7. Based on these findings, bariatric surgery did not have a major effect on ET function.

## INTRODUCTION

Obesity has emerged as a growing concern in contemporary society, defined as individuals with a body mass index (BMI) of 30 kg/m^2^ or more. The prevalence of obesity has increased among all age groups worldwide during the last three decades due to sedentary lifestyles [1]. Saudi Arabia now faces one of the highest rates of obesity and overweight individuals globally, with approximately 7 out of 10 individuals affected by these conditions [2]. Bariatric surgery has emerged as an effective treatment modality for morbid obesity. Nonsurgical management is associated with a lower percentage of weight loss (WL) and poor concomitant illness improvement [3]. Bariatric surgery is recommended for patients with a BMI exceeding 40 kg/m^2^ or those with a BMI ranging from 35 to 40 kg/m^2^, accompanied by comorbidities such as cardiovascular or pulmonary diseases, adhering to the criteria outlined by the National Institutes of Health (NIH) consensus panel [4]. As the prevalence of obesity continues to rise, bariatric surgery has gained popularity as an increasingly preferred option for individuals dealing with morbid obesity [5].

Today, Roux-en-Y gastric bypass (RYGB), sleeve gastrectomy (SG), and adjustable gastric banding are the most popular and commonly performed bariatric surgeries (BS) [6]. A key point in the debate on weight loss outcomes in bariatric surgery is patient selection. Recent guidelines from the American Society of Metabolic and Bariatric Surgery and the International Federation for the Surgery of Obesity and Metabolic Disorders (ASMBS/IFSO) showed how the criteria for choosing candidates for bariatric surgery have changed over time. Despite the variety of bariatric surgery techniques, Biliopancreatic Diversion (BPD) and Roux-en-Y Gastric Bypass (RYGB) resulted in superior weight loss than other techniques with no observed differences in diabetes resolution and adverse outcomes [7]. The Eustachian tube (ET) is an osteocartilaginous canal that connects the middle ear to the nasopharynx and is engaged in mechanisms of protection, aeration, and draining [8]. The ET is habitually closed at rest to protect the middle ear from nasopharyngeal secretions and only opens when swallowing, yawning, chewing, performing the Valsalva maneuver, and when the atmospheric pressure changes, with the help of Tensor Veli Palatini (TVPM), Levator Veli Palatine (LVPM), and salpingopharyngeus muscles [9]. However, acute weight loss following bariatric surgery can potentially lead to complications, including the reduction of soft tissue surrounding the Eustachian tube, impacting its function. This condition is known as a patulous Eustachian tube (PET), where the Eustachian tube remains constantly open [10]. Signs and symptoms associated with this condition include autophony, cacophony, and a sensation of aural fullness, all indicative of tubal dysfunction [11]. Even though symptoms of auditory changes linked to tube dysfunction are not uncommon among these patients, only a few researchers have investigated this connection. This study aimed to establish a link between signs and symptoms of Eustachian tube dysfunction following bariatric surgery in obese patients.

## MATERIAL AND METHODS

This prospective cohort study was conducted between May 2022 and December 2022, involving a total of 54 bariatric surgery cases and 157 control subjects. Inclusion criteria for the study group comprised patients of varying ages (18-60), both genders and Saudi and non-Saudi individuals who had undergone surgical obesity management. Exclusion criteria for the study group encompassed participants outside the designated time frame, age group, those utilizing non-bariatric surgical weight reduction methods, and those who did not complete the 3-month postoperative follow-up. The participants in the control group were selected randomly with the same socio-demographic characteristics, age group, and nationality status as the study group to minimize bias. Exclusion criteria for the control group included individuals who had undergone any form of weight reduction, whether surgical or non-surgical.

Data collection took place in two phases: preoperatively and at a 3-month postoperative follow-up, with a universal collection form filled out at a bariatric clinic by the authors. The form aimed to gather information on socio-demographics, pre-and postoperative weight, type of bariatric surgery, and Eustachian Tube Dysfunction Questionnaire (ETDQ-7) responses for assessing ET function. Data analysis was conducted using Microsoft Excel and the Statistical Package for the Social Sciences (SPSS), employing numbers, percentages, figures, and tables. The statistical significance of categorical variables was assessed using the Chi-square test (χ2). Data analysis was carried out by the authors and verified by a biostatistical specialist, ensuring the participants' personal information remained confidential.

## RESULTS

The study was conducted between May 2022 and December 2022, involving a total of 54 cases of bariatric surgery and 157 control subjects. Among the participants, 116 (55%) were in the age group of 18-25 years, with the majority (91.4%) not having undergone bariatric surgery (BS). Notably, the subjects who underwent BS were significantly more prevalent in the age groups >50 years, 40-50 years, and 31-40 years compared to those in the lower age groups (p<0.001). In our sample, there were 174 (82.5%) females and 37 (17.5%) males, with a significantly higher percentage of male subjects (45.9%) having undergone BS compared to females (21.3%) (p=0.002) (Table 1).

**Table 1:** Sociodemographic characteristics

			Bariatric surgery		Total	p value
			No	Yes		
Age	18-25	N	106	10	116	<0.001
		%	91.4%	8.6%	55%	
	26-30	N	9	7	16	
		%	56.3%	43.8%	7.6%	
	31-40	N	20	16	36	
		%	55.6%	44.4%	17.1%	
	40-50	N	16	13	29	
		%	55.2%	44.8%	13.7%	
	>50	N	6	8	14	
		%	42.9%	57.1%	6.6%	
Gender	Female	N	137	37	174	0.002
		%	78.7%	21.3%	82.5%	
	Male	N	20	17	37	
		%	54.1%	45.9%	17.5%	
BMI	Underweight	N	13	0	13	<0.001
		%	100.0%	0.0%	6.2%	
	Normal	N	81	0	81	
		%	100.0%	0.0%	38.4%	
	Overweight	N	40	0	40	
		%	100.0%	0.0%	18.9%	
	Obese	N	23	54	77	
		%	29.9%	70.1%	36.5%	

**Figure 1:**
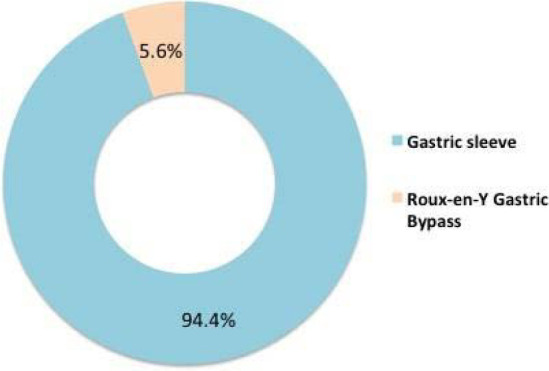
Type of bariatric surgery

In the current study, 77 subjects had a BMI >30 (classified as obese), and among them, 54 (70.1%) had undergone BS (p<0.001). Among those who underwent weight loss surgery (WLS), 51 (94.4%) had gastric sleeve surgery, and the remaining 3 (5.6%) had Roux-en-Y Gastric Bypass (RYGB) (Figure 1). The mean weight before WLS was 113.5±21.6 Kg, and after WLS, it was 92.5±20.3 Kg, showing a statistically significant difference (p<0.001) (Table 2). The mean percentage of weight loss after surgery was 45.6±18.5%.

**Table 2:** Weight loss before and after surgery (n=54)

	Mean	SD	p value
Weight before surgery	113.5	21.6	<0.001
Weight after 3 months of surgery	92.5	20.3	

The Eustachian Tube Dysfunction Questionnaire (ETDQ-7) consists of seven items that assess Eustachian tube dysfunction. The mean total ETDQ-7 in control subjects was significantly higher (11.29±5.49) compared to those who had BS (9.11±4.09), p=0.008. Except for the fourth item ("Ear symptoms when you have a cold or sinusitis"), there were no statistically significant differences in scores for the remaining six items (Table 3). When we compared the ETDQ-7 scores before and after BS, there were no statistically significant differences observed for the total ETDQ-7 score or any of the seven individual items (p>0.05) (Table 4). A repeated measured MANCOVA analysis showed that age, gender, and type of BS did not affect Eustachian tube function before and after surgery (p>0.05) (Table 5).

**Table 3:** Comparison of Eustachian Tube Dysfunction between the two groups

	Bariatric surgery	N	Mean	Std. Deviation	p value
Pressure in the ears	Yes	54	1.37	1.01	0.859
	No	157	1.40	0.82	
Pain in the ears	Yes	54	1.22	0.69	0.061
	No	157	1.48	0.91	
Feeling of ears being clogged or ‘under water	Yes	54	1.43	0.96	0.097
	No	157	1.70	1.07	
Ear symptoms with cold/sinusitis	Yes	54	1.46	1.00	0.015
	No	157	1.92	1.22	
Crackling or popping sounds in the ears	Yes	54	1.26	0.76	0.105
	No	157	1.49	0.94	
Ringing in the ears	Yes	54	1.46	1.13	0.229
	No	157	1.67	1.06	
Feeling of hearing being muffled	Yes	54	1.52	1.18	0.468
	No	157	1.64	1.06	
Total ETDQ-7 score	Yes	54	9.11	4.09	0.008
	No	157	11.29	5.49	

**Table 4:** Comparison of ETDQ-7 before and after bariatric surgery (n=54)

		Mean	Std. Deviation	p value
Pressure in the ears	Before surgery	1.11	0.42	0.071
	After surgery	1.37	1.01	
Pain in the ear	Before surgery	1.19	0.68	0.716
	After surgery	1.22	0.69	
Feeling of ears being clogged or under water	Before surgery	1.24	0.78	0.086
	After surgery	1.43	0.96	
Ear symptoms with cold/sinusitis	Before surgery	1.52	1.04	0.641
	After surgery	1.46	1.00	
Crackling or popping sounds in the ears	Before surgery	1.26	0.78	1.000
	After surgery	1.26	0.76	
Ringing in the ears	Before surgery	1.39	0.98	0.481
	After surgery	1.46	1.13	
Feeling of hearing being muffled	Before surgery	1.41	0.90	0.274
	After surgery	1.52	1.18	
Total ETDQ-7 score	Before surgery	10.73	5.24	0.218
	After surgery	10.89	5.40	

**Table 5:** Repeated-Measures MANCOVA model for ETDQ-7 difference before and after BS

Source	Type III Sum of Squares	df	Mean Square	F	p value
Intercept	592.047	1	592.047	17.071	<0.001
Age	97.432	1	97.432	2.809	0.100
Gender	.003	1	.003	.000	0.992
Type of BS	30.607	1	30.607	.883	0.352
Error	1734.040	50	34.681		

## DISCUSSION

In this current study, we examined a total of 211 participants. Among them, 116 (55%) fell within the 18-25 age group. The age range in our study is consistent with prior research, such as Kinasz *et al*., which had participants aged 19-58 years [10]. Similarly, the study by Yazici *et al*. included individuals aged 19 to 58, with a mean age of 39.32±11.09 years [9]. In our research, 82.5% (n=17) of participants were women and 17.5% (n=37) were men. In the Kinasz *et al*. study, there were 80 patients, mostly females (77) and only 3 males [10]. Similarly, in the Yazici *et al*. study, the participants consisted of 80.3% women (n=61) and 19.7% men (n=15) [9].

Our research found that most BMI measurements were in the normal range of 38.4% (n=81). In contrast, in the Kinasz *et al*. study, patients with and without symptoms in relation to pre and postoperative BMI (kg/m^2^) and pre and postoperative weight (kg) were examined. Patients with initial symptoms weighed 112 kg and had a BMI of 45 kg/m^2^, whereas those who were initially asymptomatic often weighed 117 kg and had a BMI of 47 kg/m^2^. Initial weight, postoperative weight, and pre-and postoperative BMI varied significantly between patients with and without symptoms [10]. Patients in the study of Yazici *et al*. ranged in weight from 88 to 182 kg, with an average of 127.16±20.25 kg. Patients also varied in length from 134 to 186 cm, with an average of 163.33±9.51 cm [9]. In our study, the weight before weight loss surgery (WLS) was 113.5±21.6 kg, and surgery resulted in an average weight loss of 45.6%, a statistically significant change. The most common type of weight loss surgery was gastric sleeve surgery, accounting for 94.4% of cases (n=51), and 5.6% (n=3) underwent Roux-en-Y Gastric Bypass. The study of Kinasz *et al*. included patients who underwent Roux-en-Y gastric bypass with at least 6 months of postoperative follow-up [10]. Conversely, Yazici *et al*. included bariatric surgery patients but did not specify which type [9].

We utilized the Eustachian Tube Dysfunction Questionnaire (ETDQ-7), comprising 7 questions assessing Eustachian tube dysfunction symptoms. We evaluated two groups: the first group consisted of 54 patients who underwent bariatric surgery, while the second group (the control group) comprised 157 individuals who had not undergone such surgery. Overall, the mean total ETDQ-7 in control subjects was significantly higher (11.29±5.49) compared to those who had BS (9.11±4.09), p=0.008. Notably, all six items, except for the fourth item regarding ear symptoms during colds or sinusitis, did not exhibit statistically significant differences in scores between the two groups. In another study, hearing evaluation was done by otoscopy, tonal, and vocal audiometry, and a hearing questionnaire was completed by 19 patients. Before surgery, none of the patients had any symptoms of tubal dysfunction. At the initial postoperative evaluation, postoperative data showed that 5 (26.3%) patients had symptoms associated with Eustachian tube dysfunction. 9 (47.3%) patients with a 6-month follow-up experienced tubal dysfunctional symptoms [8]. Another study used ETDQ-7 in 76 patients who underwent bariatric surgery between 2018 and 2019 and revealed 10.5% of ETD symptoms post-bariatric surgery [9]. The study by Kinasz *et al*. showed the presence of ETD in 18.75% of the patients [10]. In the current study, there was no significant difference in ETD symptoms among all 54 cases before and three months after BS, regardless of the type of BS. A few clinical cases have reported the effect of weight loss on the Eustachian tube function. The first case reported in the English literature was in 2009 when a patient developed symptoms of ear dysfunction after losing 20 kg within 3 months post laparoscopic Roux-en-Y gastric bypass surgery [12]. Munoz *et al*. case series, including 163 patients, showed a significant prevalence of patulous Eustachian tube (PET) in bariatric surgery patients (21.28 %). The prevalence of symptoms was correlated with weight loss velocity. Patients with PET experienced weight loss at an average of 48.63 kg in 12.11 months, while patients without PET experienced weight loss at an average of 39.54 kg for 16.59 months [13]. An Egyptian recent case report found that acute rapid weight loss after bariatric surgery resulted in Eustachian tube dysfunction [14]. These data contradict our findings since the average weight before BS was 113.5 kg, and the average weight after three months of BS was 92.5 kg with statistical significance. Neither of the individuals exhibited ETD or PET symptoms. On the other hand, a novel Saudi case report showed that moderate weight loss had a favorable effect on ETD [15]. A recent study examined the impact of weight loss following bariatric surgery on ET function using dynamic slow-motion video endoscopy (DSVE) and Ethylenediaminetetraacetic acid 7 (EDTA-7). These assessments were conducted before bariatric surgery and in the sixth month postoperatively. The findings of the study indicated that for the majority of patients, rapid weight loss resulting from bariatric surgery led to improvements in both DSVE imaging and the symptoms associated with ET dysfunction. However, in certain individuals, this weight loss could result in PET [16]. Eighty patients were evaluated in a recent study that revealed that 18.75% of those who underwent bariatric surgery had PET symptoms. Symptomatic patients have lower preoperative weight and BMI than asymptomatic patients [10]. This was not evaluated in the current study. Zahide *et al*. revealed that the incidence of ETD was about 10.5% in 76 patients 6 months after the bariatric surgery. Similar to our research, they used the ETDQ-7. However, they additionally assessed three parameters: autophony, ear fullness, and the perception of their breath in the ear. Compared to patients without ETD, the mean age of patients with ETD was substantially greater [9]. Consistent with the literature, the current study revealed that patients' age, gender, or the type of BS underwent had no effect on ETD pre- and post-bariatric surgery [13, 15, 17].

## CONCLUSION

The study found that the mean total ETDQ-7 score was significantly higher in control subjects compared to those who had undergone bariatric surgery. Additionally, when we compared the ETDQ-7 scores in subjects who had BS before and after surgery, no statistically significant differences were observed in the total ETDQ-7 score. Based on these findings, it appears that bariatric surgery did not have a significant impact on ET function. The authors recommend a larger study with multi-center involvement to validate the findings of the current study.

## Data Availability

Further data is available from the corresponding author on reasonable request.
